# PD-L1 expression on malignant cells is no prerequisite for checkpoint therapy

**DOI:** 10.1080/2162402X.2017.1294299

**Published:** 2017-02-21

**Authors:** Jan Willem Kleinovink, Koen A. Marijt, Mark J. A. Schoonderwoerd, Thorbald van Hall, Ferry Ossendorp, Marieke F. Fransen

**Affiliations:** a Department of Immunohematology and Blood Transfusion, Leiden University Medical Center, Leiden, the Netherlands; b Department of Medical Oncology, Leiden University Medical Center, Leiden, the Netherlands; c Department of Gastroenterology and Hepatology, Leiden University Medical Center, Leiden, the Netherlands

**Keywords:** Biomarker, checkpoint blockade, expression, immunotherapy, PD-L1, PD-1

## Abstract

Immunotherapy with PD-1/PD-L1-blocking antibodies is clinically effective for several tumor types, but the mechanism is not fully understood. PD-L1 expression on tumor biopsies is generally regarded as an inclusion criterion for this cancer therapy. Here, we describe the PD-L1-blocking therapeutic responses of preclinical tumors in which PD-L1 expression was removed from cancer cells, but not from immune infiltrate. Lack of PD-L1 expression on malignant cells delayed tumor outgrowth in a CD8^+^ T cell-mediated fashion, showing the importance of this molecule in immune suppression. PD-L1 expression was evident on myeloid-infiltrating cells in the microenvironment of these tumors and targeting stromal PD-L1 with blocking antibody therapy had additional antitumor effect, demonstrating that PD-L1 on both malignant cells and immune cells is involved in the mechanism of immunotherapeutic antibodies. Importantly, comparable results were obtained with PD-1-blocking therapy. These findings have implications for inclusion of cancer patients in PD-1/PD-L1 blockade immunotherapies.

## Introduction

The co-inhibitory receptor PD-1 and its ligand PD-L1 form a well-known immune-inhibiting axis, and engagement of PD-1 on T cells is important for maintaining peripheral tolerance and preventing over-activation of the immune system. PD-L1 expression by tumors is a powerful escape mechanism through which tumors can evade control by T cell immunity.^1^^,^^2^ The clinical relevance of this immunosuppressive pathway was emphasized by the clinical successes of PD-1 and PD-L1 checkpoint blockade therapies in a range of solid tumors.^3–6^ Beneficial outcome of PD-1 and PD-L1 therapy has been correlated with mutational burden of the cancer cells, presence of tumor-infiltrating T cells and PD-L1 expression.^1^^,^^2^^,^^7^^,^^8^ Tumor cells may upregulate PD-L1 to counteract immune attack in response to soluble mediators of T cell responses, including interferons and interleukins.^1^^,^^2^ However, various types of cancer constitutively express PD-L1, either as a result of structural alterations in the regulatory 3′ region of the PD-L1 gene,^9^ or through activation of the STAT3 and AKT pathways, which are common features of cancer.^10^^,^^11^ Although several studies have investigated PD-1 and PD-L1 interactions within tumors, the exact cellular mechanisms are not completely elucidated.^1^^,^^2^ PD-L1 can be expressed by malignant cells, but is also expressed on infiltrating cells within tumors, such as macrophages, neutrophils, endothelial cells and fibroblasts. Currently, arbitrary cut-off values for the percentage of overall PD-L1 staining in the total tumor area are used to classify tumor biopsies as positive or negative for PD-L1 expression. A recent study described a correlation between PD-L1 expression and an improved response to PD-L1 blockade in a clinical trial of multiple types of cancer patients, especially when expression was on tumor-infiltrating cells.^12^ However, no convincing proof of the relative importance of tumor-expressed versus stromal-expressed PD-L1 to therapy response exists to date. For future use as a predictive biomarker for therapeutic responsiveness, it is essential to understand the relevance of the expression pattern of PD-L1 within the tumor area. To evaluate this topic, we made use of two mouse tumor models (MC38 and CT26) on two different genetic backgrounds in which the PD-L1 gene in the cancer cells was knocked out with CRISPR-Cas9 technology. We show here that PD-L1 expressed on cancerous cells is not exclusively responsible for the therapeutic effect of PD-1/PD-L1 checkpoint blockade and that PD-L1 on infiltrating (mostly myeloid) cells contributes significantly. Our data indicate that PD-L1 expression on malignant cells is not required for successful PD-1/PD-L1-blocking therapy, which has important clinical implications regarding patient inclusion strategies for immune checkpoint blockade.

## Results and discussion

### PD-L1 is expressed on tumor cells and immune-infiltrating cells

**Figure 1.**
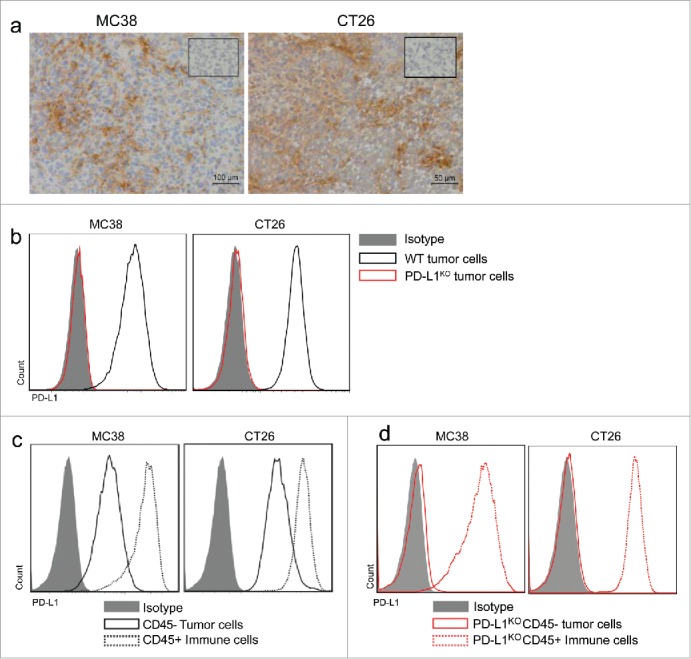
PD-L1 is expressed on tumor cells and infiltrating immune cells. (A) Immunohistochemistry for PD-L1 expression in MC38 (left) and CT26 (right) tumors. Cryosections of snap-frozen excised tumors were made 10 d after tumor inoculation and stained for PD-L1 expression (brown). Scale bars are provided in the bottom right corner. Insert shows control staining with secondary antibody only. Representative images of n = 3 tumors. (B) Flow cytometry histograms showing PD-L1 expression of MC38 tumors cells (left plot) or CT26 tumor cells (right plot). WT tumor cells (black) or PD-L1^KO^ tumor cells (red) were incubated for 48 h with IFNγ and stained for PD-L1 expression (PE-conjugated antibody) for flow cytometry analysis, using isotype-PE stained cells (gray) as controls. (C) Flow cytometry histograms for PD-L1 expression (*x*-axis, PE-conjugated antibody) of excised MC38 (left) or CT26 (right) tumors. Both tumors express PD-L1 on both tumor cells (solid black line) and on tumor-infiltrating immune cells (dotted black line). (D) Flow cytometry histograms for PD-L1 expression of excised PD-L1^KO^ MC38 (left) or CT26 (right) tumors showing lack of PD-L1 expression on PD-L1^KO^ tumor cells (solid red line) compared to infiltrating immune cells from PD-L1^KO^ tumors retained high PD-L1 expression (dotted red line).

PD-L1 expression was determined in MC38 and CT26 tumors, two widely used murine colon carcinoma models that are responsive to several types of immunotherapy including PD-L1 blockade, and are known to have a high mutational load leading to neo-antigen presentation.^13^^,^^14^ After staining sections of excised MC38 and CT26 tumors with PD-L1 antibody, we observed heterogeneous PD-L1 expression throughout the tumor, with faint expression on some cells and strong expression on clusters of other cells (Fig. 1A). Several correlation studies have been done on PD-L1 expression and prognosis, however, many are fraught with practical problems including heterogeneous expression patterns and antibody variability. ^15–17^ Unlike the majority of clinical PD-L1 biomarker assays, one assay distinguishes expression on malignant cells versus tumor-infiltrating immune cells.^12^ To dissect the role of PD-L1 expression on tumor versus non-tumor cells, we created PD-L1-deficient variants of MC38 and CT26 tumor cells using CRISPR-Cas9 technology. Complete PD-L1 knockdown was confirmed by flow cytometry after interferon-gamma (IFNγ) stimulation *in vitro* (Fig. 1B). In order to specify which cells in MC38 and CT26 tumors express PD-L1, we inoculated mice with WT and PD-L1^KO^ tumor cells and analyzed the cell suspensions of excised tumors by flow cytometry. We determined that in WT tumors, PD-L1 expression was present on CD45-negative tumor cells, but also strongly on CD45^+^ immune infiltrate (Fig. 1C). PD-L1^KO^ tumors still contained this strong PD-L1 expression on CD45^+^ immune cells (Fig. 1D). A recent study in other mouse tumor models reported that PD-L1 deficiency affected tumor cell viability and proliferation.^18^ However, the absence of PD-L1 on MC38 and CT26 tumor cells did not hamper *in vitro* proliferation (Fig. S1).

### PD-L1 on cancer cells suppresses CD8^+^-mediated immune control

**Figure 2.**
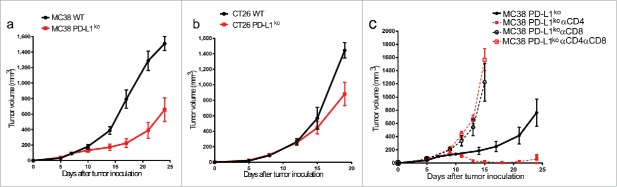
PD-L1 on cancer cells suppresses CD8^+^ T cell-mediated immune control. Tumor outgrowth curves of (A) MC38 and (B) CT26 tumors growing in B6 and BALB/c mice, respectively. Mice were inoculated with either WT tumor cells (black circles) or PD-L1^KO^ tumor cells (red squares) on day 0, and tumor volume was followed in time. The graphs show the group average tumor volume and contain pooled data from three independent experiments with 20–22 mice (MC38) or two independent experiments with 12–15 mice (CT26). Volumes of mice that had to be killed due to local ethical guidelines of maximal tolerated tumor size were afterwards counted as last measured volume. Line was stopped when more than 50% of mice in group had died due to tumor burden. By Student's *t* test, differences in WT vs. PD-L1KO MC38 tumor volumes are statistically significant on day 14 (*p* < 0.01) and days 17, 21 and 24 (*p* < 0.001). Differences in WT vs.PD-L1^KO^ CT26 tumor volumes are statistically significant on day 19 (*p* < 0.01). (C) Tumor outgrowth curves of PD-L1^KO^ MC38 tumors either without treatment (solid black line), with CD4^+^-depleting antibody (solid red line, closed squares), with CD8^+^-depleting antibody (dotted black line, open circles) or with both CD4^+^- and CD8^+^-depleting antibodies (dotted red line, open squares). Tumors were inoculated on day 0, depleting antibodies were injected periodically from day 5 on, and tumor volume was followed in time. Volumes of mice that had to be killed due to local ethical guidelines of maximal tolerated tumor size were afterwards counted as last measured volume. Line was stopped when more than 50% of mice in group had died due to tumor burden. Differences in tumor volume compared to untreated PD-L1^KO^ MC38 tumors are statistically significant (*p* < 0.05, Student's *t* test) for αCD4 treatment on days 13 through 25 (all *p* <0.01), for αCD8 treatment on days 13 (*p* < 0.05) and 15 (*p* < 0.01), and for αCD4^+^ αCD8 treatment on days 11, 13 and 15 (all *p* < 0.001).

In order to determine whether the lack of PD-L1 expression on tumor cells alters tumor growth characteristics *in vivo*, we injected immunocompetent mice with either WT or PD-L1^KO^ tumor cells. Although both WT and PD-L1^KO^ tumor cells formed established tumors within one week after inoculation, subsequent outgrowth of PD-L1^KO^ MC38 tumors was significantly slower compared to WT MC38 tumors (Fig. 2A). A similar delay in tumor outgrowth was observed for PD-L1^KO^ CT26 tumor cells versus WT CT26 cells after injection in immunocompetent BALB/c mice, albeit less pronounced than in the MC38 model (Fig. 2B). Our observation of identical growth rates in culture but delayed outgrowth of PD-L1^KO^ cells in mice suggested that the immune system inhibited growth of PD-L1^KO^ tumors. To test this, we treated mice-bearing PD-L1 proficient or deficient MC38 tumors with CD4^+^- or CD8^+^-depleting antibodies and followed tumor outgrowth. Depletion of CD8^+^ T cells or depletion of both CD8^+^ and CD4^+^ T cells completely abrogated the delayed outgrowth of PD-L1^KO^ tumors, confirming our hypothesis on the tumor-eradicating effector function of cytotoxic T cells in this model (Fig. 2C). In contrast, depletion of only CD4^+^ T cells enhanced tumor clearance, suggesting that CD4^+^ T cells in this model most likely represent regulatory T cells, as has been published in several settings (Fig. 2C).^1^^,^^2^ These findings of T cell-dependent retardation of tumor outgrowth of PD-L1^KO^ cancer cells suggest active control by CD8^+^ T cells, which are inhibited by PD-L1 expressed on tumor cells.

### Tumor microenvironment is not altered by PD-L1 deficiency of cancer cells

**Figure 3.**
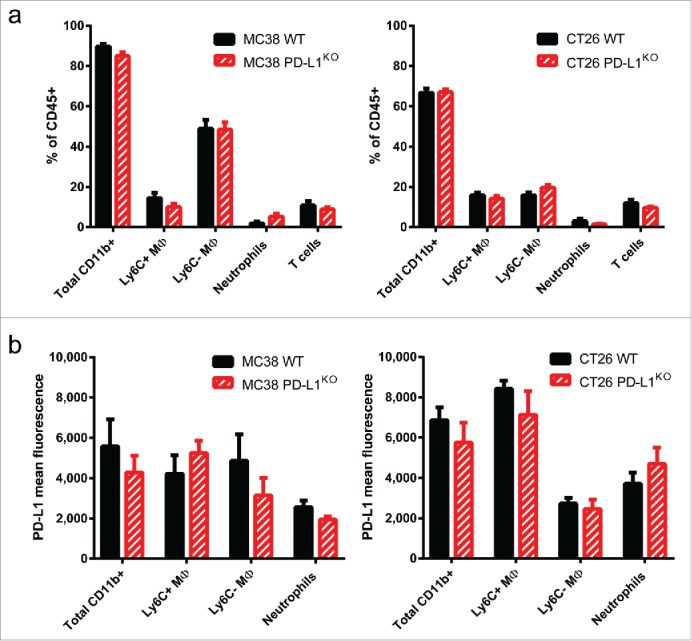
Tumor microenvironment is not altered by PD-L1 deficiency of cancer cells. (A) Bar graphs showing the percentages of immune cell subsets infiltrating MC38 tumors (left) or CT26 tumors (right) comparing WT tumors (black bars) with PD-L1^KO^ tumors (red bars). Established tumors were excised and processed for flow cytometry analysis, using 7-AAD to exclude dead cells. Gating strategies are as follows; Total CD11b = CD45^+^ CD11b^+^ cells; Ly6C^+^ MΦ = CD45^+^ CD11b^+^ F4-80^+^ Ly6G^−^ Ly6C^+^; Ly6C^−^ MΦ = CD45^+^ CD11b^+^ F4-80^+^ Ly6G^−^ Ly6C^−^; Neutrophils = CD45^+^ CD11b^+^ Ly6G^+^ and T cells: CD45^+^ CD11b^−^ CD3^−^. None of the differences are statistically significant. (B) Bar graphs showing the geometric mean fluorescence for PD-L1 expression (PE-conjugated antibody) on the myeloid immune subsets described in (A). None of the differences are statistically significant according to the Student's *t* test.

We observed high levels of PD-L1 expression on immune cells in WT tumors, which may further contribute to the CD8^+^ T cell-mediated control of tumor outgrowth (Fig. 1B). It has recently been published that tumor-infiltrating T cells can have a profound effect on the myeloid composition of the tumor microenvironment.^19^^,^^20^ Therefore, we analyzed the immune infiltrate composition in WT versus PD-L1^KO^ tumors in the MC38 and CT26 model. Surprisingly, no significant quantitative differences in T cell infiltrate of WT versus PD-L1^KO^ tumors were found (Fig. 3A). Furthermore, neither total CD11b^+^ myeloid infiltrate was altered, nor were the Ly6C^+^ and Ly6C^−^ macrophages and neutrophils. Moreover, PD-L1 expression levels on tumor-infiltrating myeloid cells were not significantly altered by the lack of PD-L1 expression on the malignant cells in either tumor model (Fig. 3B). These results indicate that the composition of the myeloid immune infiltrate is not strongly influenced by PD-L1 expression on the malignant cells.

### PD-L1 blockade is still effective against PD-L1^KO^ tumors

**Figure 4.**
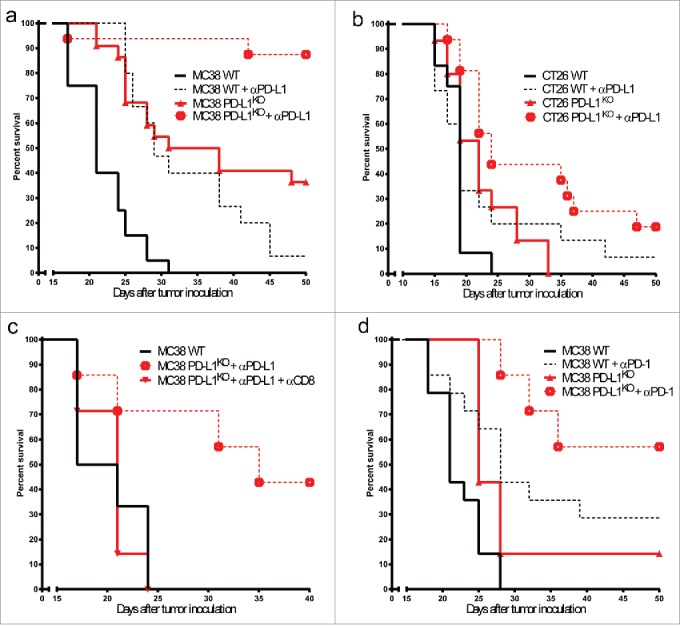
PD-L1 blockade is still effective against PD-L1^KO^ tumors. Survival curves of mice bearing (A) MC38 or (B) CT26 tumors either left untreated (solid lines) or treated with PD-L1 blocking antibody (dotted lines), comparing WT tumors (black lines) to PD-L1^KO^ tumors (red lines). Antibodies were given on days 5, 8 and 11. For both tumor models, tumor outgrowth curves of individual mice are provided in Figs. S2 and 3. In both tumor models, Log-rank analysis shows statistically significant differences (*p* < 0.05) between survival of each group compared to the others. (C) Survival curves of B6 mice bearing untreated WT MC38 tumors (solid black line), αPD-L1 treated PD-L1^KO^ MC38 tumors (dotted red line), or αPD-L1 treated PD-L1^KO^ MC38 tumors with CD8^+^ depletion (solid red line). The effect of CD8^+^ depletion on the survival of αPD-L1 treated PD-L1^KO^ MC38 tumor-bearing mice is statistically significant (*p* < 0.05) using the Log-rank test. (D) Survival curves of B6 mice-bearing WT (black lines) or PD-L1^KO^ (red lines) MC38 tumors, either left untreated (solid lines) or treated with PD-1-blocking antibody on days 5, 8 and 11 (dotted lines). Log-rank analysis shows statistically significant (*p* < 0.05) differences in survival between untreated and PD-1-blockade-treated mice for both WT and PD-L1^KO^ tumors. In all graphs, survival refers to the time before reaching the maximally allowed tumor volume of 2,000 mm^3^.

To test whether the therapeutic efficacy of PD-L1 blockade is solely based on alleviating immune suppression by PD-L1 expression on cancer cells, we examine treatment efficacy with PD-L1-blocking antibody in mice carrying WT or PD-L1^KO^ tumors. Remarkably, outgrowth of PD-L1^KO^ MC38 tumors was even further decreased by therapeutic PD-L1 blockade, leading to complete eradication of most tumors and long-term survival of nearly 90% of animals (Fig. 4A and Fig. S2 for individual tumor growth curves). A less striking but similar additional effect of PD-L1 blockade in PD-L1^KO^ tumors was found in the CT26 model (Fig. 4B and Fig. S3 for individual tumor growth curves). These data indicate that PD-L1 expression on non-tumor cells also contribute to immune evasion and thereby tumor outgrowth. Depletion of CD8^+^ T cells during PD-L1 blockade of PD-L1^KO^ MC38 tumors returned tumor growth rates to the level of untreated WT tumors, showing that non-tumor cell expression of PD-L1 contributes to the inhibition of effector CD8^+^ T cell responses (Fig. 4C). A similar effect was observed with therapy with a different PD-L1-blocking antibody clone (data not shown). To test whether the absence of PD-L1 on cancer cells also influenced the therapeutic efficacy of PD-1 blockade, we treated mice-bearing WT or PD-L1^KO^ MC38 tumors with PD-1-blocking antibody. Again, there was an additional strong treatment effect by PD-1 blockade of PD-L1^KO^ tumors, showing that the treatment effect was effectively mediated through blocking the inhibiting PD-1/PD-L1 interactions on non-tumor cells (Fig. 4D).

In parallel with a recently published study by Noguchi et al.,^21^ our findings concur with the clinical study by Herbst et al.,^12^ which describes that patients with PD-L1 expression on tumor cells and/or immune infiltrate have a more favorable outcome of therapeutic PD-L1 blockade. Our experimental models provide a mechanistic basis underlying these clinical observations, by showing that PD-L1 expression on both tumor cells and on tumor-infiltrating immune cells inhibits CD8^+^ T cell responses against the tumor. Complete responses were found in many, but not all animals. This can probably be explained by the fact that PD-1/PD-L1 is only one of several immune-inhibiting mechanisms. PD-1 also binds to PD-L2, but its expression is less pronounced than PD-L1 expression.^22^ Several other compensatory interactions, such as upregulation of LAG3, TIM-3 and other immune checkpoint molecules have been found in tumors, indicating the diversity and flexibility of the tumor–immune cell interactions.^1^ Therefore, combinatorial therapies with immune-modulating antibodies, based on multiple molecular interactions within the tumors, have more potential.^1^^,^^2^ Nonetheless, the great efficacy of PD-1/PD-L1 axis blockade as monotherapy in curing mice from aggressive tumors indicates a dominant role. Altogether, our work shows that malignant cells and tumor-infiltrating immune cells jointly suppress CD8^+^ T cell responses by PD-L1 expression, and that tumors lacking PD-L1 expression on malignant cells can be efficiently treated by PD-1/PD-L1 axis blockade therapy.

## Material and methods

### Mice and cell lines

C57BL/6 mice were obtained from Harlan Laboratories–ENVIGO (The Netherlands) and BALB/c mice were purchased from Charles River (France) and housed under specified pathogen-free conditions in animal facilities of the Leiden University Medical Center. All animal experimentations were approved by and according to guidelines of the Dutch Animal Ethical committee. MC38 and CT26 cells (kindly provided by Mario Colombo) were cultured in IMDM medium (Lonza) containing 8% Fetal Calf Serum (Greiner), 100 IU/mL penicillin/streptomycin (Gibco), 2 mM glutamin (Gibco) and 25 µM 2-mercaptoethanol. Cell lines were mycoplasma and MAP-tested before the start of experiments.

### Tumor inoculation and immunohistochemistry

Tumors were inoculated by subcutaneous injection in the right flank of 500,000 MC38 cells or 100,000 CT26 cells in 100 µL PBS. Tumor outgrowth was measured by caliper in three dimensions, until mice had to be sacrificed due to tumor burden, according to local ethical guidelines. For immunohistochemistry, established tumors were excised on day 10 and 5 µm frozen sections were fixed using ice-cold acetone and blocked in 0.3% hydrogen peroxidase (MERCK) in methanol. Next, slides were stained for PD-L1 (clone 10F.9G2, BioLegend), secondary biotin-conjugated rabbit anti-rat IgG (Boster Biological Technology) and Vectastaincomplex (Vector labs). Color was developed using DAB+ reagent (DAKO) and nuclei were counterstained with Myers Haematoxylin (Merck). Slides were mounted using Entellan (Merck). Photos were taken using an Olympus DX51 light microscope and Olympus cellSens software.

### CRISPR/Cas9

For CRISPR/Cas9-knockout of PD-L1, an online CRISPR Design Tool (crispr.mit.edu) was used to design two gRNA sequences for exon 1 of the mouse *cd274* gene encoding the PD-L1 protein (gRNA #1 = GTATGGCAGCAACGTCACGA, gRNA #2 = GCTTGCGTTAGTGGTGTACT) and each gRNA was cloned into a gRNA cloning vector (Addgene 41824). Next, MC38 or CT26 tumor cells were transfected with these two gRNA plasmids (2 μg/plasmid) and with Cas9 WT (Addgene 41815), using the Lipofectamine 2000 protocol (ThermoFisher). Cells were then stimulated for 48 h with 20 IU/mL interferon-gamma to upregulate PD-L1 on WT cells and stained with PE-labeled PD-L1 antibody for FACS-sorting of PD-L1^KO^ cells.

### In vitro proliferation assay

3,000 cells of each tumor cell line were seeded, and after 24, 48 or 72 h cells were pulsed with 1 µM ^3^H and analyzed 15 h later.

### Treatments

Tumor-bearing mice were treated on day 5, 8 and 11 after tumor inoculation by intraperitoneal injection of 200 µg PD-L1-blocking antibody (clone 10F.9G2, BioXCell) or peritumoral subcutaneous injection of 50 µg PD-1-blocking antibody (clone RMP1-14, BioXCell). T cells were depleted by intraperitoneal injection of 50 µg depleting antibody (clone 2.43 for CD8^+^, clone GK1.5 for CD4^+^, both in-house production) on day 5 after tumor inoculation. Complete depletion was confirmed on the following day in peripheral blood by flow cytometry, and mice were screened periodically and re-injected when T cell populations started returning in peripheral blood.

### Flow cytometry

Cell surface staining was performed using the following antibodies: CD8α (clone 53–6.7), CD4^+^ (clone L3T4), CD3ε (clone 145-2c11), CD11b (clone M1/70), F4-80 (clone BM8), CD45.2 (clone 104), Ly6G (clone 1A8), Ly6C (clone HK1.4), PD-L1 (clone MIH5). For analysis of the tumor microenvironment, tumor-bearing mice were sacrificed, and perfused with 20 mL of PBS/EDTA (2 mM) to eliminate blood contamination of tumor material. Tumors were cut into small pieces with scalpels, incubated with 2.5 mg/mL Liberase TL (Roche) for 20 min at 37°C and single-cell suspensions were made using 70-µm cell strainers (BD Biosciences). Fc-receptors were blocked with 10% normal mouse serum before antibody staining. Dead cells were excluded based on 7-AAD (Invitrogen). Samples were analyzed with LSRII cytometer (BD) using FacsDIVA software (BD) and FlowJo software (Tree Star).

### Statistical analysis

GraphPad Prism 7 software was used for all statistical analyses. The means of two groups were compared using the Student's *t* test, and survival differences in Kaplan–Meier curves were analyzed by Log-rank test. Differences were considered statistically significant at *p* <0.05.

## Supplementary Material

KONI_A_1294299_supplemental_data.zip
