# Supplementary material for: PD-L1 expression on malignant cells is no prerequisite for checkpoint therapy
Source: Oncoimmunology. 2017 Feb 21;6(4):e1294299. doi: 10.1080/2162402X.2017.1294299 (PMC5414865; doi:10.1080/2162402X.2017.1294299)
Supplement: KONI_A_1294299_supplemental_data.zip [file koni-06-04-1294299-s001.zip › KONI_A_1294299_s02.pdf]

Supplemental Figure 1

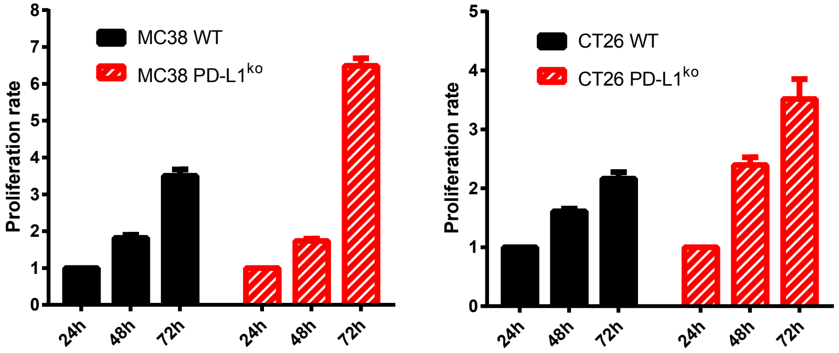

Supplementary Figure S1. In vitro proliferation plots of MC38 (left) and CT26 (right) tumor cells, either WT (black) or PD-L1^{KO} (red), based on the 3H incorporation assay. Cells were seeded and pulsed with ^{3}H at 24, 48 or 72 hours and analyzed 15h later.
