# Supplementary material for: PD-L1 expression on malignant cells is no prerequisite for checkpoint therapy
Source: Oncoimmunology. 2017 Feb 21;6(4):e1294299. doi: 10.1080/2162402X.2017.1294299 (PMC5414865; doi:10.1080/2162402X.2017.1294299)
Supplement: KONI_A_1294299_supplemental_data.zip [file koni-06-04-1294299-s001.zip › KONI_A_1294299_s03.pdf]

Supplemental Figure 2

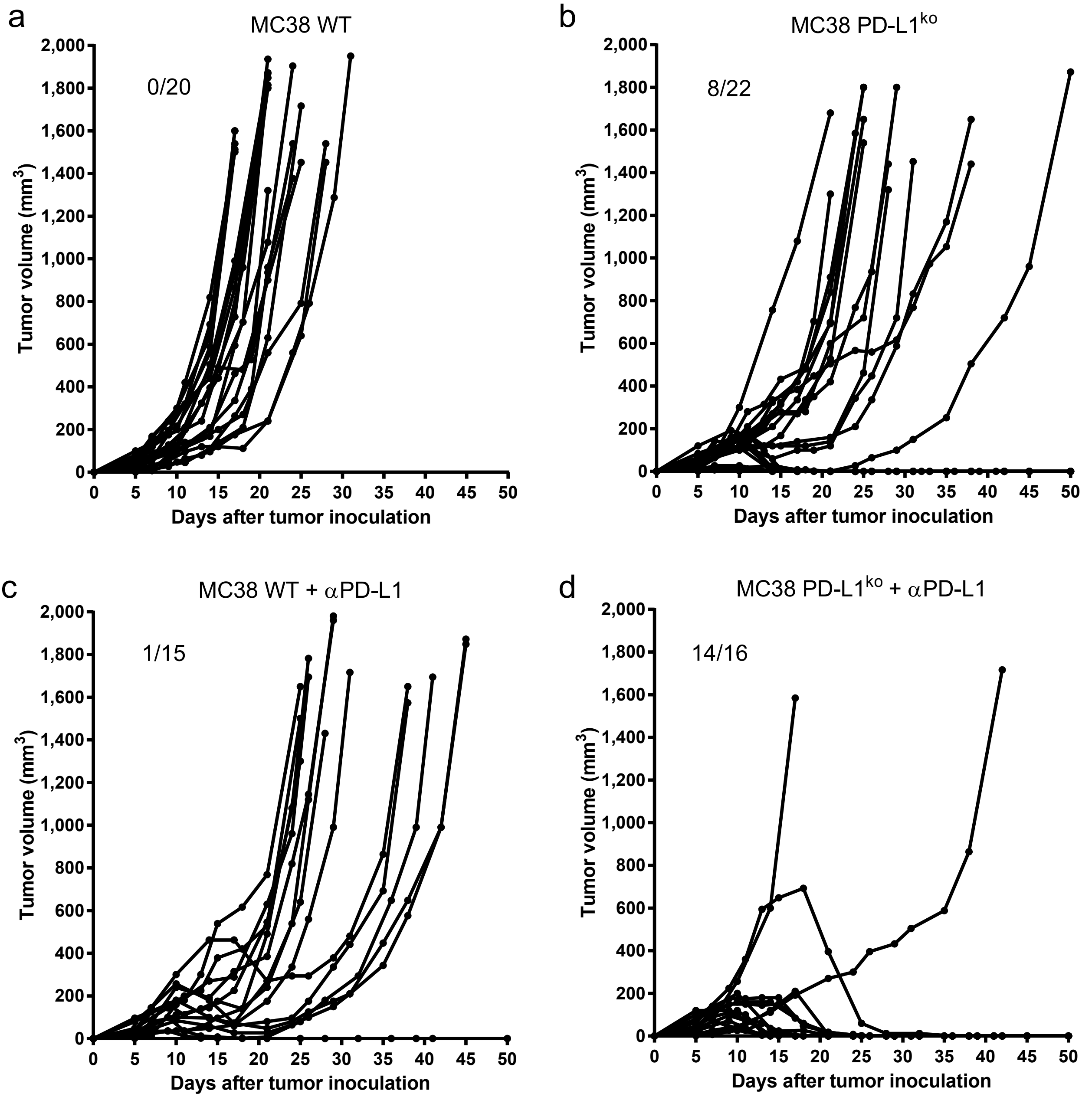

Supplementary Figure S2. MC38 tumor outgrowth curves of individual B6 mice bearing **(A)** untreated WT tumors, **(B)** untreated PD-L1^{KO} tumors, **(C)** WT tumors treated with PD-L1 blockade or **(D)** PD-L1^{KO} tumors treated with PD-L1 blockade. Graphs correspond to survival curves in Figure 4A. Each line represents the tumor of an individual mouse. The fraction of mice that fully cleared the tumor is indicated in the graph.
